# The Effect of Motivation on Physical Activity among Middle and High School Students

**DOI:** 10.3390/sports12060154

**Published:** 2024-05-30

**Authors:** Hélio Antunes, Ana Rodrigues, Bebiana Sabino, Ricardo Alves, Ana Luísa Correia, Helder Lopes

**Affiliations:** 1 Department of Physical Education and Sport, Faculty of Social Sciences, University of Madeira, 9020-105 Funchal, Portugal; h.antunes@staff.uma.pt (H.A.); anajar@staff.uma.pt (A.R.); ricardo.alves@staff.uma.pt (R.A.); anacorreia@staff.uma.pt (A.L.C.); 2 Centre for Tourism Research, Development, and Innovation (CITUR), 9020-105 Funchal, Portugal; 3 Research Center in Sports Sciences, Health Sciences, and Human Development (CIDESD), 5001-801 Vila Real, Portugal; 4 Higher School of Education, Polytechnic Institute of Beja, 7800-295 Beja, Portugal; bebiana.sabino@ipbeja.pt; 5 Sport Physical Activity and Health Research & Innovation Center (SPRINT), 3030-329 Coimbra, Portugal

**Keywords:** motivation, formal physical activity, informal physical activity, students, middle school, high school

## Abstract

The study addressed two main objectives: (i) to investigate disparities in motivation dimensions regarding extracurricular physical activity and (ii) to identify the influence of motivation on time spent in formal and informal physical activity. A sample of 704 adolescents (56% girls) from middle (46%) and high school (54%), with an average age of 14.88 ± 2.52, was assessed for different motivation dimensions using the Questionnaire of Motivation for Sports Activities (QMSA). Additionally, participants were categorized based on extracurricular physical activity practice. Multivariate analyses and multiple linear regressions were conducted to examine the effect of physical activity type on motivation dimensions and identify predictors of time spent in formal and informal physical activities, respectively. Results indicated that motivation varied significantly with extracurricular physical activity practice (*p* < 0.05), with students involved in extracurricular activities being more motivated. Sex and age differences were observed, with boys showing higher motivation in certain dimensions (achievement status (*p* < 0.001); group activity (*p* = 0.027); contextual (*p* = 0.004); technical improvement (*p* = 0.012) and older participants having lower scores in all dimensions. The influence of family and friends was a significant predictor only for boys in formal physical activity (*p* = 0.039). In terms of time spent in physical activity, group activity was a predictor for informal activities (*p* < 0.001), while technical improvement was a predictor for formal activities (*p* < 0.001), with notable sex differences. These findings underscore the importance of considering sex- and age-specific motivations when promoting physical activity among adolescents.

## 1. Introduction

The documented health advantages of physical activity (PA) during adolescence are manifold and well established in the domain of public health, encompassing enhanced cardiorespiratory and muscular fitness and improved bone and cardiometabolic health [1]. Resistance and weight-bearing exercises are crucial for developing muscle mass and attaining optimum bone density, which is essential for musculoskeletal health in later life [2]. In addition to its physical health advantages, there is a growing body of evidence supporting the cognitive, psychological, and social benefits associated with PA [3,4]. Research indicates that a considerable segment of health advantages continues to persist into adulthood [5,6]. To harness these benefits, the Canadian Physical Activity Guidelines for Children and Youth [7], Australian Physical Activity Guidelines for Children and Young People [8], Physical Activity Guidelines for Americans [9], and World Health Organization [10] advise that adolescents aim for moderate-to-vigorous intensity PA for a minimum of 60 min each day. Despite the obvious health benefits of PA, evidence suggests a decline in PA during adolescent years [2,3,11,12,13,14]. This decline is concerning, especially considering that physical inactivity is acknowledged as a global pandemic [15].

Physical inactivity, often coupled with obesity—a known precursor of numerous adverse outcomes—substantially contributes to the development of non-communicable diseases [16], including diabetes [17], functional impairment [18], cancer [19,20], and cardiovascular diseases [21,22].

Physical activity is a fundamental premise for the mental health of young students, as it contributes to reducing tension and stress [23,24]. Various studies have shown that psychological distress, depression, and anxiety disorders negatively correlate with engagement in moderate physical activities [25]. Moreover, intense physical activity leads to even lower levels of academic stress, enhancing motivation and self-confidence among students [26,27].

Given the health risks associated with physical inactivity, it is recommended to intensify efforts aimed at gaining a deeper understanding of health and well-being during adolescence, along with exploring potential factors influencing engagement in PA [28,29]. Among these factors, motivation plays a crucial role in predicting intentional behaviors, such as engaging in PA during physical education and adulthood [30], and a cornerstone for sustaining PA, especially among adolescents who are navigating through a period of significant physical and psychological development [31]. This aligns with the findings of a study conducted on Vizcaya University students, highlighting that motivation significantly influences the student’s engagement in PA [32]. In terms of motivation and gender, studies demonstrate nuanced differences in the motivations and needs for sports participation between boys and girls [33,34]. Empirical evidence suggests that girls are less active than boys [2], and lower PA motivation was observed among girls than in boys in adolescents [35]. Moreover, various research shows that girls indicate a decline in enjoyment motives [36], physical fitness [37], and social motives [38], and an increase was observed only in appearance motives [37,39]. Scraton [40] emphasized how school sports and physical education perpetuate gender stereotypes and traditional socialization of gender roles, noting boys’ emphasis on competition and performance improvement in sports contrasted with girls’ inclination towards social interaction and friendships. A plethora of research supports this idea, highlighting athletic boys’ inclination towards satisfaction of winning and competition [41,42], while girls prioritize fun and making friendships [43,44]. Furthermore, boys commonly favor sports modalities characterized by physical confrontation and intense competition, exemplified by football and rugby [40,45]. Conversely, girls typically prioritize sociability and dedication to game-like and sporting activities [41,46]. Interventions designed to enhance motivation for PA, particularly in school settings, have shown promise in increasing enjoyment motives, particularly among girls [47].

These variations in motivation for PA observed between boys and girls, particularly in their participation in extracurricular sports, underscore the necessity for a comprehensive comprehension of gender-based motivations [31]. This understanding is crucial for developing targeted intervention programs to suit individual preferences and needs aimed at habit development, enhancing adolescents’ PA levels, and mitigating associated health risks linked with physical inactivity.

Although it is widely recognized that motivation influences participation in physical activity (PA), little research has been conducted on comparing the same population across different practice contexts. This comparison may provide unique insights into how various environments and structures of physical activities can affect adolescents’ motivation.

Thus, the primary objectives of this study are threefold: (i) to analyze the association between gender and age and motivational dimensions for physical activity; (ii) to investigate disparities in motivational dimensions between participants who exclusively attend physical education classes at school and those who are involved in extracurricular physical activities, such as school sports or federated sports; and iii) to understand the influence of different motives on boys’ and girls’ formal and informal sports practice. By addressing these objectives, the study aims to contribute valuable insights toward developing effective gender-specific intervention strategies tailored to promote active lifestyles among adolescents.

## 2. Materials and Methods

### 2.1. Study Design and Participants

The study is cross-sectional in nature, with all subjects evaluated only once at the beginning of the school year. It included 704 adolescents of both sexes (306 boys and 398 girls) with a mean age of 14.88 ± 2.52 years. This study includes participants from five schools (middle and high) in the research project “Physical Education in Schools from the Autonomous Region of Madeira” [48]. Participants were informed of the aims of the study, and written informed consent was obtained from their legal guardians. Individuals with pathologies and/or special educational needs were excluded. Only those who completed the questionnaire were included in the study.

The study received ethical approval from the Scientific Committee of the Faculty of Physical Education and Sport, University of Madeira (reference: ACTA N.77-12.04.2016). The study was also approved by the Regional Secretary for Education and Culture and by the headmasters of the participating schools.

The assessments were carried out by physical education and sports graduates. The assessors received specific training as follows: (i) they received instructions and demonstrations regarding how to apply the questionnaires and the interview, and how the accelerometers worked; (ii) the members of the field team practiced with each other; and (iii) the team took part in a pilot study for all the variables. Fifteen adolescents (8 boys and 7 girls) aged between 16 and 18 were assessed twice, one week apart. The pilot study indicated good to acceptable test–retest reliability for all the assessments (interclass correlation coefficient between 0.797 and 0.999).

A single evaluation session was held, lasting approximately 30 min, during which the students completed the questionnaire.

### 2.2. Instruments

#### 2.2.1. Motivation

To assess motivation in sports activities, we employed the Questionnaire of Motivation for Sports Activities (QMSA), validated by Serpa and Frias [43], which consists of 30 items describing reasons for engaging in sports activities. These items were grouped into the following dimensions:

Achievement status: this dimension is related to the desire to achieve recognition and status through sports practice. The items that compose it include seeking to win (item 3), receiving awards (item 14), feeling important (item 21), being known (item 25), reaching a higher sporting level (item 23), overcoming challenges (item 26), competing (item 20), being recognized and having prestige (item 28).

Enjoyment: this dimension refers to the pleasure and fun associated with sports practice. The items include doing something one is good at (item 12), being with friends (item 2), making new friendships (item 11), having fun (item 29), traveling (item 5), and releasing tension (item 13).

Group activity: this dimension refers to the desire to participate in sports activities that involve social interaction and teamwork. The items include working as a team (item 8), team spirit (item 18), belonging to a group (item 22), learning new techniques (item 10), taking action (item 17), and having something to do (item 16).

Contextual: this dimension refers to the physical and social environment in which sports activity occurs. The items include the influence of coaches (item 27), enjoyment in using facilities and sports equipment (item 30), and experiencing strong emotions (item 7).

Physical fitness: This dimension refers to the importance of maintaining good physical condition through sports practice. The items include staying in shape (item 6), being in good physical condition (item 24), exercising (item 15), and having a reason to leave the house (item 19).

Technical improvement: this dimension is related to the desire to improve technical skills and sports performance. The items include releasing energy (item 4) and improving technical abilities (item 1).

Influence of family and friends: this dimension refers to the influence of family members and friends on motivation for sports practice. The item includes the influence of family or friends (item 9).

All items on the QMSA were rated on a 5-point Likert scale, with the following meanings: 1—Not important; 2—Slightly important; 3—Important; 4—Very important; 5—Extremely important. It should be noted that items 20 and 26 have reverse scoring (1 = 5, 2 = 4, 5 = 1). QMSA scoring is obtained as follows: for each dimension, the sum of the respective items is calculated and then divided by the number of items (average of items).

The reliability of the instrument was calculated using Cronbach’s α value, showing moderate to high internal consistency in the subscales: “Achievement status” (α = 0.9); “Enjoyment” (α = 0.8); “Group Activity”, “Contextual”, and “Physical Fitness” (α = 0.7); “Technical Improvement” (α = 0.6).

#### 2.2.2. Physical Activity

##### Extracurricular Physical Activity (EPA)

Participation in EPA was self-reported through the question, “Do you practice any type of sporting physical activity in addition to physical education?” (Yes/No). If so, indicate in what context, as a school sports athlete or as a federated athlete. Subsequently, the participants were categorized into three groups: (1) OPEd—subjects whose only organized physical activity is physical education classes; (2) SS—participants who, in addition to physical education, take part in school sports; and (3) S—adolescents registered as athletes in federations. The weekly duration of formal and informal physical activity was assessed using a questionnaire on a scale of 1 (never) to 8 (>7 h a day).

### 2.3. Data Analysis

Descriptive statistics such as mean and standard deviation were used to characterize the sample with respect to the variables under study. The study employed Student’s *t*-test to verify the existence of significant differences between genders, and utilized Pearson’s correlation to check correlations between age and different dimensions of motivation. The Kolmogorov–Smirnov test was used to test statistical normality. Multivariate analysis of variance, controlling for age and sex, was used to examine the effect of EPA on the different dimensions of motivation. Multiple linear regression was used to determine the influence of the different dimensions of motivation and age on time spent in formal and informal physical activity. Statistical analyses were performed using SPSS version 29.0 statistical software for Windows (SPSS Inc., Chicago, IL, USA). A 5% significance level was used.

## 3. Results

Most participants were female (56.5%) and high school students (54.0%). The motivation dimension had higher values, and the achievement status dimension was lower.

Significant differences were identified between boys and girls regarding all dimensions of motivation (*p* < 0.05), except for the “influence of family and friends” (*p* > 0.05), with boys presenting higher values on average. On the other hand, there was a negative association between age and all dimensions of motivation (−0.108 < r < −0.237), except for technical improvement (*p* > 0.05).

The majority of the sample had physical education as their only organized physical activity and low levels of formal and informal physical activity (Table 1).

Multivariate analysis of variance controlling for age and sex showed an effect of EPA on the various dimensions of motivation (*p* < 0.05). The effects varied between small and medium. Participants with OPEd showed lower average values in all dimensions of motivation (Table 2).

Sex influenced the following dimensions: (i) achievement status (*p* < 0.001; η^2^ = 0.033); (ii) group activity (*p* = 0.027; η^2^ = 0.007); (iii) contextual (*p* = 0.004; η^2^ = 0.012), and (iv) technical improvement (*p* = 0.012; η^2^ = 0.009). In all dimensions, boys showed higher values than girls.

There was also an effect of age on the dimension’s achievement status (*p* < 0.001; η^2^ = 0.016); group activity (*p* < 0.026; η^2^ = 0.033); contextual (*p* < 0.001; η^2^ = 0.028) and influence of family and friends (*p* < 0.001; η^2^ = 0.021). Older participants had lower mean scores in all dimensions.

Multiple linear regression was used to determine the influence of motivation and age on informal and formal physical activity. It appears that 9.2% (boys) and 5.9% (girls) of the variation in informal physical activity is explained by the group activity dimension. The technical improvement dimension represents 6.4% (boys) and 11.9% (girls) of the variation in formal physical activity. The influence dimensions of family and friends (boys) and contextual (girls) equally explain 1.3% and 1.9% of the variation in formal physical activity (Table 3).

## 4. Discussion

Enjoyment emerges as a primary motivational factor among third-cycle and secondary school students when engaging in PA. Literature consistently highlights the absence of enjoyment as a predominant driver behind the discontinuation of sports participation [49,50]. Indeed, researchers do not universally agree on the significance of enjoyment as a determining factor in the motivation of children and young individuals to participate in sports; however, some stories support this assertion [51,52,53]. On the other hand, there are studies in which this evidence is not verified [54,55]. Physical fitness and group activity were also highly valued dimensions by the students who took part in the study, contrasting with the lesser consideration given to the achievement status dimension. It was found that most students did not engage in any kind of extracurricular PA. This finding aligns with the result corroborated by Kilpatrick, Hebert [56], who assert that despite the recognized benefits associated with a healthy lifestyle, sedentary lifestyles and a lack of PA are still a reality among the student population.

### 4.1. Effect of Gender and Age on the Dimensions of Motivation for Physical Activity and Sport

The results of the study pointed to differences in motivation for PA based on gender and age of the students, consistent with findings in existing literature [57,58,59]. Specifically, it was found that boys exhibited higher motivation scores in the “achievement status”, “enjoyment”, “group activity”, “contextual”, “physical fitness”, and “technical improvement” dimensions.

Several studies investigated motivation for PA have similarly indicated differences between the sexes, with boys tending to be more competitive and motivated by achievement status factors [60]. This suggests that boys place greater emphasis on competition and athletic performance, potentially promoting sporting success that will enable them to attain social recognition [59].

Boys’ motivation for “physical fitness” and “technical improvement” was higher than girls’. This result is in line with the literature, which underscores that boys’ motivation to take part in sports is based on a combination of intrinsic factors, such as personal challenge and improving technical skills, which they value as a way of achieving personal goals [54,61]. Additionally, extrinsic factors, such as competition and the quest for victory, play a role in motivating boys’ participation [62]. These results underscore the importance of providing opportunities and resources for young men to cultivate and refine their technical skills in extracurricular physical activities, thus enhancing their motivation and sustained commitment to participation.

Also, “enjoyment” was significantly more valued by boys, as several studies addressing this topic indicate [63,64,65]. Boys’ intrinsic motivation to practice PA is the predominant factor, as they pursue pleasure and strive for overall health benefits [37,39,66,67,68].

The contextual dimension was valued more highly by boys, consistent with findings from certain studies [69]. However, the literature shows that aspects related to the influence of peers, family, and teachers can be either inhibitors or facilitators of girls’ motivation and loyalty to practicing EPA [70]. This highlights the importance of considering not only gender differences, but also the specific relational context in which the students are situated.

Regarding the “group activity” dimension, the results obtained in the study contradict the various studies that show that girls are more motivated to take part in physical and/or sporting activities in groups [60,71]. Some studies suggest that girls display heightened motivation to interact and socialize with others, especially during adolescence, when social relationships play a fundamental role in emotional and psychosocial development [58]. Girls seem to be more motivated by factors related to group activities and value the presence of friends and teammates more as a reason for practicing and continued engagement in sports. Furthermore, group activities are also seen as influencing mental health among adolescents, contributing to their emotional well-being, and this is also an aspect valued by girls [72].

The differences observed between the results of the study and the literature may be related to contextual variables such as the individual characteristics of the participants characteristics, past experiences in group activities, and the perception and level of motor proficiency. Specifically, regarding motor proficiency, because boys perceive themselves as possessing specific skills or talents that distinguish them from group settings, they may be more motivated to participate. On the other hand, if girls feel less assured about their motor proficiency or social skills in group contexts, this may decrease their motivation.

There is a negative association between age and the following dimensions: achievement status, enjoyment, group activity, contextual, physical fitness, and influence of family and friends. Older participants showed lower mean scores across these dimensions.

With regard to the achievement status dimension, the fact that older students (secondary school) had lower scores may suggest a decline in motivation associated with pursuing success in extracurricular physical activities decreases as students age [54,73]. As adolescents advance in educational level and consequently in age, their orientation towards achieving success may diminish. This can be influenced by a variety of factors, including changes in the school environment, personal development, and individual priorities. As students encounter more rigorous academic challenges and social pressures and develop different interests and identities, their motivation for social promotion through PA may wane.

Although fun is considered one of the main reasons for children and young people to engage in sports, the study results show that its importance diminishes as they grow older. The literature suggests that this trend may be related to adults placing more value on winning than on having fun [74,75]. Consequently, children and young people grow up in an environment where the highest regard is given to athletes who achieve the best results.

Younger students, on the other hand, may value group activities more due to the need for social belonging and the desire to interact with their peers [76]. However, as they advance in education (from the third cycle to secondary school), their priorities and interests may evolve, influencing their motivation to participate in group activities.

The contextual dimension of motivation was also affected by age, with older students showing lower average scores. Although the specific literature on this dimension is limited, research on the social and emotional development of adolescents, such as the study by Eccles and Roeser [77], may provide additional insights into the factors that influence contextual motivation over time.

The physical fitness dimension is also less valued by older students. This finding is supported by [78,79]. This may be related to the fact that students in higher grades are more demotivated to engage in sports due to increasing academic pressure and other responsibilities that consume their time and energy. Additionally, physical and emotional changes during adolescence, such as self-criticism and fluctuating self-esteem, can also reduce interest in participating in physical activities.

Finally, the influence of age on the influence of family and friends dimension indicates that older students tend to attribute less importance to this dimension in their motivation to practice extracurricular physical activities. Although specific studies related to influence of family and friends are not abundant, some older ones [80,81,82] suggest that the social environment, including the influence of family and friends, plays a significant role in the development of motivation in young athletes and can vary with age as adolescents undergo different stages of development and sporting experiences.

### 4.2. Effect of Extracurricular Physical Activity (OPEd, SS, S) on the Various Dimensions of Motivation

Motivation for EPA among young people is influenced by variety of factors, such as pleasure, competence, intrinsic motivation, physical fitness, learning new skills, fun, success, and status, and it is crucial to consider the context in which PA occurs, as the literature recognizes different motives for federated sports activities or informal physical activities [60,83].

In this study, it was found that students with different types of PA (i) who only have physical education (OPEd); (ii) who practice school sport (SS); and (iii) who practice federated sport (S) have significantly different motivations. Thus, students who only had physical education classes (OPEd) had lower average values in all dimensions of motivation. This suggests that participation in extracurricular activities (school sports and federated sports) fosters higher motivation compared to those exclusively attending PE lessons (OPEd) at school.

These results are corroborated by some studies which have shown that participation in school sports or other extracurricular activities can promote a greater sense of belonging [84], intrinsic motivation [72,85,86,87], and self-esteem among students. Furthermore, from another perspective, there is evidence that participation in extracurricular activities may also be associated with a greater willingness to learn and improved mental health among young people [88]. These findings highlight the importance of extracurricular physical activities as an effective means of promoting students’ motivation and well-being [89]. Offering a variety of activities in extracurricular settings can cater to the diverse needs and interests of students, thereby enhancing motivation and commitment to PA or sport [90]. Therefore, the results of the study could have significant implications for the planning of physical education programs and extracurricular activities in schools, highlighting the importance of promoting a school culture that values and supports students’ active participation in physical activities beyond school hours.

### 4.3. The Influence of Different Motives on Boys’ and Girls’ Formal and Informal Sports Practice

Studies in the literature suggest that certain reasons influence the amount of PA people do [91,92]. The results found in this study point to significant differences between boys and girls in the motivational factors that influence the time spent practicing sports, both formal and informal. The group activity dimension emerges as a predictor of informal physical activity for both boys and girls. The dimension of group activity emerges as a predictor of informal physical activities for both genders, suggesting that opportunities for social interactions and group participation motivate both sexes to dedicate more time to informal physical activities, consistent with research highlighting the importance of social relationships in motivating PA [91,93]. Conversely, concerning formal physical activities, such as training and competitions in school sports or federated sports, both boys and girls are motivated by the search for technical improvement, and this variable is a predictor of a longer period of practice. This may reflect the importance attributed to the development of specific skills and sporting evolution within the structured context of formal sport [94]. Notably, the influence of family and friends emerges as a significant predictor only for boys in practicing formal physical activity, suggesting that social support and peer influence may have a more direct impact on boys’ participation in organized and structured sports, which is in line with research depicting the role of family and friends in promoting PA among young people [81,95].

It is imperative to acknowledge the differences between the sexes in motivation to practice sports and to highlight the need for differentiated approaches to promoting PA among boys and girls. Furthermore, it is suggested that intervention programs and policies to promote PA should be sensitive to the specific needs and preferences of students of different genders, with the aim of maximizing commitment and participation over time.

## 5. Study Limitations

While this study provides valuable insights into the motivation and participation in EPA among adolescents, several limitations must be acknowledged. First, the reliance on self-reported data to measure EPA may introduce recall and response biases despite efforts to mitigate these biases with standardized and validated questionnaires. We recognize that objective methods, such as accelerometers, could complement self-reported data in future research.

The cross-sectional design used in this study prevents the determination of causal relationships between the factors studied. However, it establishes a solid foundation for future longitudinal research that can explore the evolution of motivation and participation over time.

The sample, consisting of adolescents from five schools in the Autonomous Region of Madeira, provides a relevant snapshot, but generalizing the results to other regions or cultural contexts should be engaged in with caution. Additional studies in different geographical and cultural contexts would be beneficial to validate and expand our findings.

The exclusion of contextual factors such as family support, sports infrastructure, and school policies are a limitation we acknowledge. Nevertheless, this study intentionally focused on specific motivational variables to provide a clear starting point for future research that may incorporate these additional factors.

Despite these limitations, the study significantly contributes to the understanding of adolescent motivation for EPA.

## 6. Conclusions

Based on the study objectives aimed to analyze whether the reasons for engaging in sports varied by sex and age, as well as to investigate differences in motivation dimensions concerning the type of sports activity performed (physical education, school sports, or federated sports) and to identify the influence of motivation on the time spent in formal and informal physical activity, several important conclusions emerged.

Firstly, it was found that the motivations for sports participation differ significantly between boys and girls in all dimensions except for the “influence of family and friends” dimension, with boys showing lower average values across all dimensions.

Multivariate analysis of variance showed a significant effect of EPA on various motivation dimensions, indicating that participants solely engaged in physical education showing lower average values across all motivation dimensions.

Multiple linear regression analysis revealed that the group activity dimension significantly accounted for variation in informal physical activity, especially for boys. On the other hand, the technical training dimension was a significant predictor of formal physical activity, with higher explained variances for girls. The influence of family and friends, alongside contextual dimension equally explained variations in formal physical activity, albeit with sex-specific differences.

These conclusions highlight the intricate interplay between motivation, demographic factors, and the type of PA adolescents engage in. Understanding these dynamics is crucial for developing effective interventions and policies aimed at promoting PA and overall well-being among young people.

## Figures and Tables

**Table 1:** Sample description.

	Variables	Total
Sociodemographic	Men *n* (%)	306 (43.5%)
	Women *n* (%)	398 (56.5%)
	Age (years)	14.88 ± 2.52
	Middle school *n* (%)	324 (46.0%)
	High school *n* (%)	380 (54.0%)
Motivation	Achievement status	2.80 ± 0.53
	Enjoyment	3.88 ± 0.71
	Group activity	3.79 ± 0.77
	Contextual	3.41 ± 0.85
	Physical fitness	3.84 ± 0.74
	Technical improvement	3.70 ± 0.82
	Influence of family and friends	3.46 ± 1.17
Extracurricular Physical Activity	OPEd	430 (61.1%)
	SS	92 (13.1%)
	S	182 (25.8%)
PA Informal (h/week)	<1 h	230 (32.7%)
	Between 1 and 2 h	127 (18.0%)
	Between 2 and 3 h	132(18.8%)
	Between 3 and 4 h	68 (9.7%)
	Between 4 and 5 h	35 (4.9%)
	Between 5 and 6 h	37 (5.2%)
	Between 6 and 7 h	30 (4.3%)
	>7 h	45 (6.4%)
PA Formal (h/week)	None	430 (61.1%)
	Between 1 and 2 h	22 (3.1%)
	Between 2 and 3 h	63 (8.9%)
	Between 3 and 4 h	45 (6.4%)
	Between 4 and 5 h	26 (3.7%)
	Between 5 and 6 h	30 (4.3%)
	Between 6 and 7 h	28 (4.0%)
	>7 h	60 (8.5%)

OPEd—subjects whose only organized physical activity is physical education classes; SS—participants who, in addition to physical education, take part in school sports; S—adolescents registered as athletes in federations; PA—physical activity.

**Table 2:** Multivariate analysis of variance of EPA in motivation, controlling for age and sex.

Dimension of Motivation	Extracurricular Physical Activity			F	η^2^	PostHoc
	OPEd (*n* = 430)	SS (*n* = 92)	S (*n* = 182)			
Achievement status	2.70 ± 0.51	2.97 ± 0.51	2.95 ± 0.55	9.78 *	0.028	OPEd < SS; OPEd < S
Enjoyment	3.74 ± 0.72	4.12 ± 0.63	4.08 ± 0.69	14.79 *	0.042	OPEd < SS; OPEd < S
Group Activity	3.60 ± 0.74	4.16 ± 0.69	4.05 ± 0.71	23.27 *	0.065	OPEd < SS; OPEd < S
Contextual	3.17 ± 0.82	3.82 ± 0.79	3.77 ± 0.77	30.35 *	0.083	OPEd < SS; OPEd < S
Physical Fitness	3.67 ± 0.74	4.18 ± 0.65	4.08 ± 0.64	22.93 *	0.064	OPEd < SS; OPEd < S
Technical Improvement	3.50 ± 0.78	4.04 ± 0.85	4.01 ± 0.75	28.26 *	0.077	OPEd < SS; OPEd < S
Influence of Family and Friends	3.29 ± 1.15	3.92 ± 1.05	3.64 ± 1.19	8.757 *	0.025	OPEd < SS; OPEd < S

Values are means (standard deviations ± SD) and number and proportions (%) for categorical data. Partial Eta-square (effect size): 0.01 small, 0.06 medium, 0.14 large. * *p* < 0.001.

**Table 3:** Multiple linear regression.

Dependent Variable	Sex	Independent Variable	R^2^	ß	t	*p*
Informal Physical Activity (Hours. Week) (*n* = 704)	Boys (R^2^ = 9.2%)	Group activity	9.2%	0.303	4.277	<0.001
	Girls (R^2^ = 5.9%)	Group activity	5.9%	0.242	3.956	<0.001
Formal Physical Activity (Hours. Week) (*n* = 274)	Boys (R^2^ = 7.7%)	Technical training	6.4%	0.212	3.600	<0.001
		Influence of family and friends	1.3%	0.122	2.068	0.039
	Girls (R^2^ = 13.8%)	Technical training	11.9%	0.156	2.948	0.003
		Contextual	1.9%	0.272	5.128	<0.001

## Data Availability

The data presented in this study are available on request from the corresponding author. The data are not publicly available due to privacy and ethical restrictions.
